# A question of (perfect) timing: A preceding head turn increases the head-fake effect in basketball

**DOI:** 10.1371/journal.pone.0251117

**Published:** 2021-05-12

**Authors:** Andrea Polzien, Iris Güldenpenning, Matthias Weigelt

**Affiliations:** Department of Sport & Health, Psychology and Movement Science, Faculty of Science, Paderborn University, Paderborn, NRW, Germany; University of Texas at San Antonio, UNITED STATES

## Abstract

In many kinds of sports, deceptive actions are frequently used to hamper the anticipation of an opponent. The head fake in basketball is often applied to deceive an observer regarding the direction of a pass. To perform a head fake, a basketball player turns the head in one direction, but passes the ball to the opposite direction. Several studies showed that reactions to passes with head fakes are slower and more error-prone than to passes without head fakes (head-fake effect). The aim of a basketball player is to produce a head-fake effect for as large as possible in the opponent. The question if the timing of the deceptive action influences the size of the head-fake effect has not yet been examined systematically. The present study investigated if the head-fake effect depends on the temporal lag between the head turn and the passing movement. To this end, the stimulus onset asynchrony between head turn, and pass was varied between 0 and 800 ms. The results showed the largest effect when the head turn precedes the pass by 300 ms. This result can be explained better by facilitating the processing of passes without head fake than by making it more difficult to process passes with a head fake. This result is discussed regarding practical implications and conclusions about the underlying mechanism of the head–fake effect in basketball are drawn.

## Introduction

The ability to anticipate the forthcoming actions of others is crucial in many competitive sports, in which athletes predict the actions of team members and/or opponents under high time pressure. Action anticipation in sports, however, can be hampered by the deliberate use of deceptive actions [for a review on deceptive actions in sports, see 1]. Following Jackson and colleagues [2], deceptive actions can be distinguished into two categories: actions with the aim to disguise true intentions and those with the aim to deceive the opponent. The former refers to the minimization of that kind of movement information, which is helpful to predict a particular action. In martial arts, for example, a roundhouse kick is often performed for as long as possible like a front kick, so that it becomes difficult for an opponent to identify the type of the kick [3]. The latter refers to the use of misleading information. The head fake in basketball is a paradigmatic example for such a deceptive action in which action-relevant and action-irrelevant information are presented (almost) simultaneously, in order to deceive the opponent about the actors’ true action intention. That is, during the execution of the head fake, a basketball player turns the head to one side and passes the ball to the opposite side. Thereby, the aim of a basketball player is to deceive the opponent about the throwing direction, so that the pass to a team member can be completed successfully. Several studies showed that participants react slower and more error-prone to the direction of a pass with a head fake compared to a pass without a head fake, signifying the so-called *head-fake effect* [4–6].

The impact of the head-fake effect seems to rely on the automatic processing of the head orientation of the player, which cannot be suppressed [6]. A recent study shows that the head-fake effect is based on the automatic processing of the head orientation, but not on the (otherwise socially important) gaze information [7]. In contrast to our earlier studies [e.g., 4, 6], we now refer to head orientation instead of gaze direction as task-irrelevant, interfering stimulus feature. The head-fake effect in basketball has proven to be very robust and can even be observed after extensive practice [5], and in high-level basketball experts [8]. Moreover, the head-fake effect has been found with static as well as dynamic images and with simple (i.e., keypress) as well as complex (i.e., whole body movement) responses [9]. There are some further aspects, which have an impact on the size of the head-fake effect: The head-fake effect is reduced, when the working memory of participants is taxed by another task in a dual-task scenario (e.g., counting backwards by three) [10]. Anodal transcranial direct current stimulation (tDCS) over the dorsolateral prefrontal cortex (DLPFC) also reduces the head-fake effect, but not catodal stimulation [11]. Furthermore, the frequency with which the head fake is being presented modulates the head-fake effect in the way that the effect decreases the more often the head fake is used [4, 12]. This aspect is of high practical relevance for real-sport scenarios.

Certainly, when a basketball player uses a head fake, his/her aim is to produce a head-fake effect for as large as possible in the opponent. In this context, an interesting aspect for real sports scenarios, which has not been systematically investigated so far, is the timing of the deceptive action (i.e., the temporal sequencing of movement parts or the whole movement). For the head fake in basketball, the (optimal) temporal lag between the head turn and the pass initiation may be a critical parameter for the success of the deception. Thereby, the question is, if different temporal lags between head turn and pass initiation produce head-fake effects of different magnitudes. To answer this question, the present study made use of the Posner Cueing paradigm [13, 14].

In cueing experiments, participants are instructed to respond to a target, which is preceded by a cue. The cue signals the location where the target stimulus is presented with a certain probability. The standard finding for non-informative cues is, that responses to targets at cued locations are faster and less error-prone than to targets at un-cued locations [13]. Spatial cueing has often been examined with non-social cues [15, 16]. For some time, however, the spatial cueing paradigm has also been used to investigate the influence of another person’s head or gaze orientation (both representing strong social cues) on the processing of a subsequent, peripherally presented target [17, 18].

The time course of the cueing process is often investigated by varying the stimulus onset asynchrony (SOA) between the presentation of the social cue and the target. Friesen and Kingstone [19], for example, used simple line drawings of a face to examine the effect of gaze orientation on responses to a target letter. To this end, the centrally presented face was first shown with blank eyes. After 680 ms the eyes were filled with pupils (cue) either looking straight ahead or to the left or the right side. After the SOA of 105 ms, 300 ms, 600 ms, or 1005 ms, the target letter was presented to the left or right side of the face, while the face and pupils remained on the screen. Participants were informed that the gaze direction was not predictive for the target location. In a localization task, participants were instructed to indicate the side on which the target appeared for as fast and accurately as possible. Results revealed better performance in cued as compared to un-cued conditions for every SOA up to 600 ms, signifying a gaze-cueing effect. For the SOA of 1005 ms, the gaze-cueing effect was gone. Thus, the gaze-cueing effect emerged rapidly (i.e., for an SOA of 105 ms), and was present for only a relatively short time period (i.e., it disappeared by 1005 ms) [19; for similar findings see 20].

Using non-predictive social and non-social cues, Langdon and Smith [21] presented either a head turned to the left or right (social cue head orientation), a head looking straight ahead with gaze oriented to the left or to the right (social cue gaze orientation), and arrows pointing to the left or right (non-social cue arrow orientation). Moreover, neutral cues were used, which contained no directional information. Before the cue, a pre-cue, which also contained no directional information was presented. Pre-cue and cue were displayed in the center of the screen and, after a variable SOA, an asterisk (target) was presented on the left or right side of the cue. Participants were instructed to respond to the onset of the target. For social cues, no cueing effect was observed at the shortest SOA of 100 ms, but significant head- and gaze-cueing effects were observed for SOAs between 200 ms and 800 ms [21, Exp. 1 and 2]. However, the results for the SOA 800 ms were ambiguous. In Experiment 6, SOAs between 200 ms and 1200 ms were used and no cueing effects for social cues (gaze cues) could be observed for SOA longer than 600 ms. Interestingly, the authors conducted a cost-benefit analysis to examine the relative contribution of visual attention shifts, and non-attentional automatic priming. This analysis was grounded on the assumption that automatic priming leads to faciliatory effects and emerge faster compared to costs, which are caused by attentional shifts. Langdon and Smith [21] concluded that gaze cues triggered priming effects at the SOA 200, whereas additional costs of attention shifts could be observed with SOAs of 300 ms or longer. Moreover, the authors found no reduction in the congruency effects between SOA 400, 600, and 800 (Exp. 2).

These results from cueing studies imply that social cues (e.g., gaze direction, head orientation) might modulate the processing of a subsequent target depending on the lag between both stimuli. Accordingly, the aim of the present study is to examine the optimal temporal lag between the head turn and pass initiation for the head-fake effect in basketball. To this end, a cueing experiment with static images of a basketball player with novice participants was conducted. The SOA varied between 0 and 800 ms (in steps of 100 ms) between head turn (i.e., cue; left vs. right) and passing movement (i.e., target; pass with or without head fake to the left or to the right). Targets depicting a pass with a head fake and without a head fake occurred equally often. The head orientation in the cue always matched the head orientation in the target, and thus, was not predictive for the pass direction. Participants were asked to indicate the pass direction by pressing one of two different keys on a computer keyboard.

It is important to note here that this experimental design involves a change in the head-fake paradigm exploited and therefore, is somewhat different from our previous studies on the head-fake effect [6, 12]. These previous studies revealed a perceptual origin of the head-fake effect when head turn and passing action are presented simultaneously. In this case, the different directional information conveyed by the head turn (task-irrelevant cue) and the passing action (task-relevant cue) cause a conflict, which must be solved before the task-relevant stimulus feature (i.e., pass direction) can be further processed [6]. If the cue (i.e., preceding head turn) in the present study does not trigger any additional processes, this effect of perceptual interference should nevertheless be observable and produce a head-fake effect, which is comparable to our previous studies. However, based on the study by Langdon and Smith [21] mentioned above, we assume the preceding head turn (i.e., task-irrelevant cue) to modulate the head-fake effect in the following manner: The previously presented head turn initially activates the corresponding response. If a target without a head fake occurs, the primed response could immediately be executed, which results in fast reactions in the congruent condition. If, however, a target with a head fake occurs, the (wrong) primed response needs to be cancelled before the correct response can be initiated. Referring to Langdon and Smith [21], these non-attentional priming effects should emerge with an SOA of 200 ms seconds. For an SOA 300 and larger, additional effects of an attention shift to the side, which is indicated by the head turn should come into play. If the subsequently presented target contains no head fake, the attention is already on the right side and the pass direction can be processed directly. If a target with a head fake occurs, the attention is on the wrong side and must be re-oriented to the other side, before the (task-relevant) pass direction can be processed. Those cueing and priming effects caused by the head turn should be reduced or even eliminated at SOA 800 [21].

As mentioned above, our previous studies have shown that the head-fake effect is very robust and only marginally decreases with extensive practice [5]. Even expert basketball players cannot inhibit the processing of the head fake and are in general similarly affected by the deception like novices [8]. At the same time, when running a larger research program to investigate deceptive actions in sports over an extended period of time, experts become a rare test population and we refrain from testing experts for as long as we do not see the immediate need arising from the research question. We think that the time-course of the cueing/compatibility effects in novices might similarly shed light on the underlying mechanisms of the head-fake effect and provides relevant information for optimal performance in basketball.

## Method

### Participants

An *a priori* sample-size analysis using G*Power 3 [22] was conducted to determine the required sample size. For the interaction in our 2 x 9 repeated measures design, results suggested a sample size of at least *N* = 16 participants (given *f* = 0.25, *α* = 0.05, 1-*β* = 0.9). In total, twenty-six participants took part in the experiment. One participant was excluded due to basketball experience and one due to technical problems. Moreover, one participant was excluded due to high error rates. The remaining twenty-three participants (11 females, 2 left-handed, mean age = 24.1 years, *SD* = 2.1) had no special expertise in basketball. Participants reported normal or corrected to normal vision and were naïve with regard to the purpose of the study. Each participant gave written informed consent before the experiment. Participants took part voluntarily and did not receive course credits or financial reward. The study was conducted in accordance with the seventh revision (Fortaleza) of the 1964 Declaration of Helsinki by the World Medical Association (WMA). Moreover, this research was reviewed and approved by the Ethics Committee of Paderborn University.

### Apparatus and stimuli

**Fig 1:**
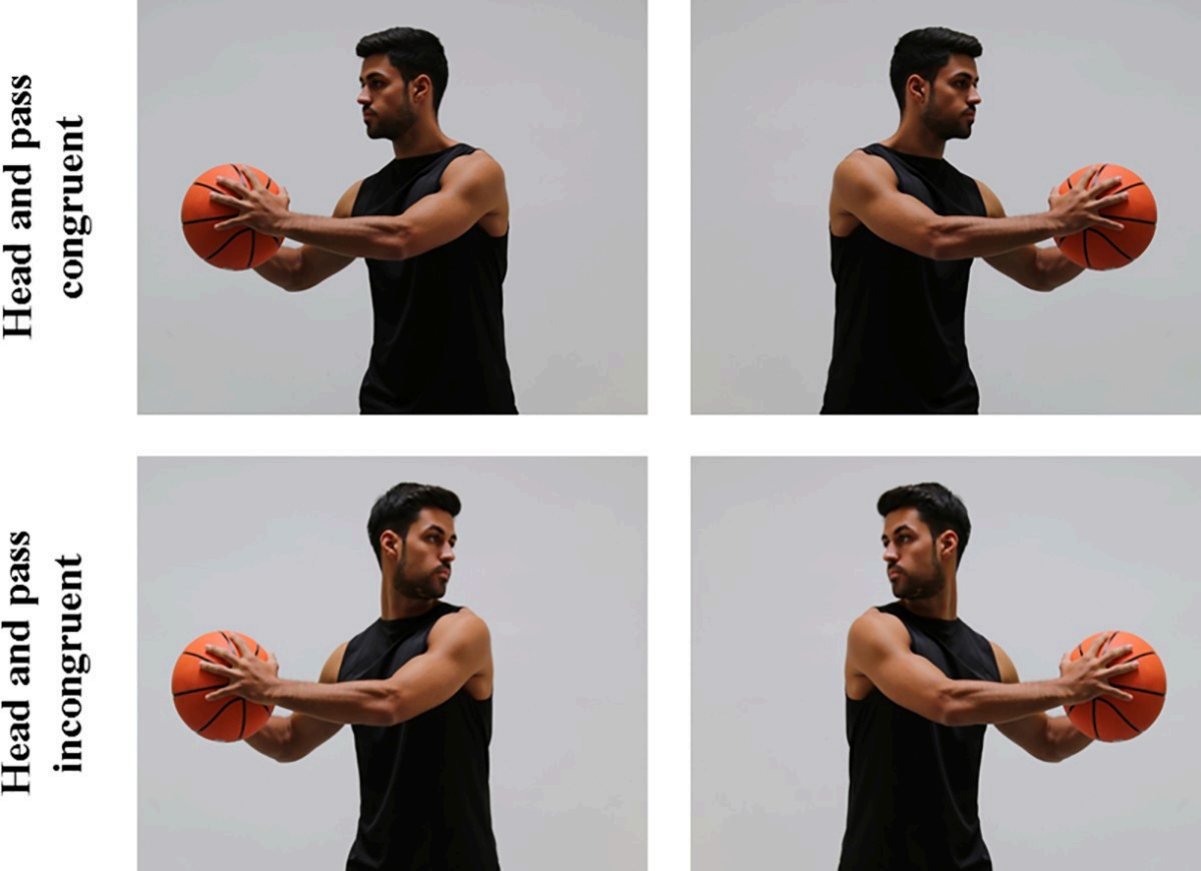
The four different targets used in the experiment.

The stimulus-set consisted of seven static images of a male basketball player, who was photographed from a front perspective. In one picture, the athlete stood in an initial position, head facing the camera and a basketball in front of the body. In two images, the head was turned to the left or the right, respectively, but the basketball was still in front of the body. In addition, there were four pictures (Fig 1), in which the head and the arms with the ball were turned to the side as if the athlete would execute a pass. These stimuli served as targets and were either congruent (i.e., head and pass to the same side) or incongruent (i.e., head and pass to different sides). The stimuli had a size of 24 x 19.5 cm (2553 x 2069 pixels) and were displayed on a 22” monitor. For presentation of the stimuli, the software “Presentation” (version 20.0, Neurobehavioral Systems) was used. As response buttons, the keys “a” and “ä” of a German keyboard were used for responses with the index finger of the left and right hand, respectively.

### Procedure

**Fig 2:**
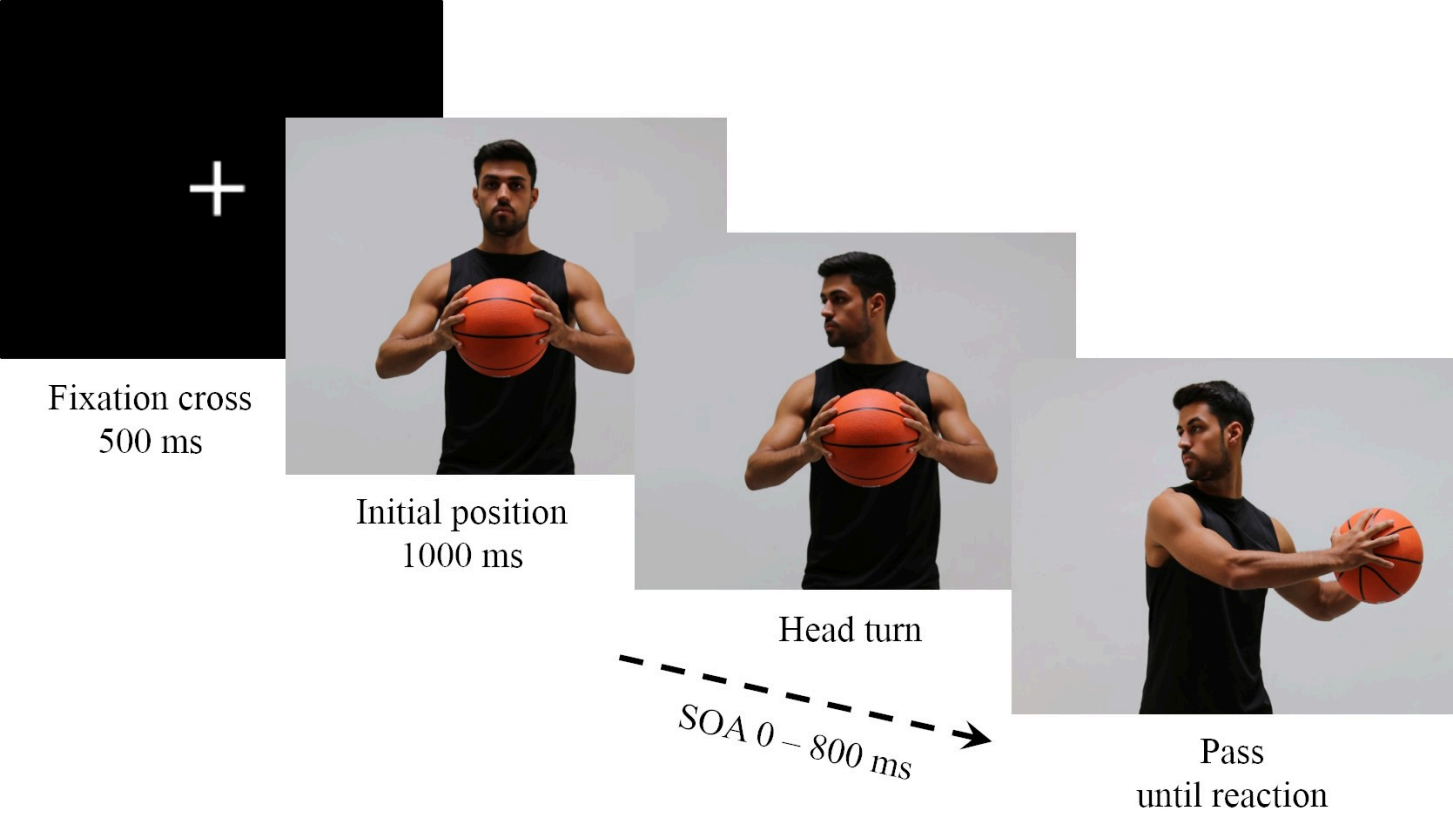
Sequence of a trial.

Participants sat approximately 60 cm in front of the monitor and were instructed to respond to the pass direction of the basketball player as fast and as accurately as possible by pressing the left or right response button. One trial consisted of a fixation cross (500 ms), the basketball player in the initial position (1000 ms), the head turn to one side (social cue; presentation duration in dependence of SOA), and the target with the basketball player executing the pass (until response; see Fig 2). The stimulus-onset asynchrony (SOA) between the head turn, and the pass was varied between 0 and 800 ms, in steps of 100 ms. In the SOA 0 condition, no preceding head turn was presented. In total, 36 conditions were used: Head and pass congruent to the left, head and pass congruent to the right, head left with pass right (i.e., head fake left) and head right with pass left (i.e., head fake right) in combination with the nine different SOA. After a correct response was given, a blank screen was shown for 2000 ms before the next trial started. In case of an incorrect response, the word “Fehler” (German word for “error”) was displayed on the monitor for 500 ms. In the beginning, participants carried out 36 practice trials (one trial for each condition), in order to get familiar with the task. Afterwards, 720 experimental trials were randomly presented (each condition 20 times) in four blocks of 180 trials each. Between each block, participants could take a rest.

### Data analysis

Reaction times (RT) below 100 ms and above 1500 ms (0.04%) as well as wrong answers (1.03%) were excluded from RT analysis. The mean errors (ER) in percent were calculated from incorrect responses. Mean RT were submitted to a repeated measures ANOVA with the factors *type of pass* (pass with or without head fake) and *SOA* (0 ms, 100 ms, 200 ms, 300 ms, 400 ms, 500 ms, 600 ms, 700 ms, and 800 ms). In case of a violation of the sphericity assumption, results were corrected according to Greenhouse-Geisser. For multiple comparisons, the alpha value was Bonferroni-Holm corrected and the corrected *p*-values are reported.

## Results

### Reaction times (RT)

**Fig 3:**
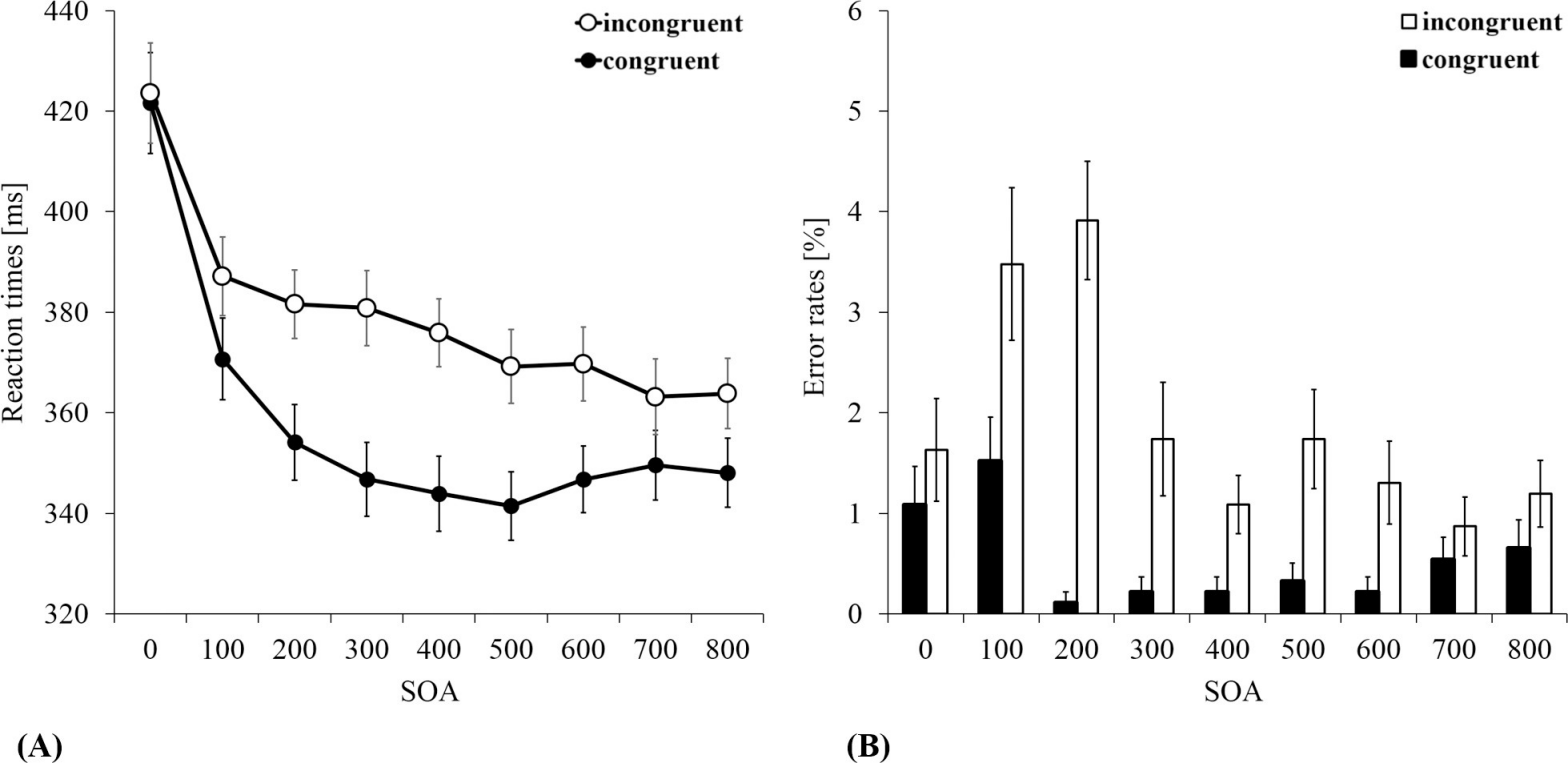
Results. Mean reaction times ± SE (A) and mean error rates ± SE (B) for the pass without a head fake (blank circles/columns) and the pass with a head fake (black circles/columns) as a function of SOA.

Mean reaction times are illustrated in Fig 3A. Results showed a significant main effect for *type of pass* [*F*(1, 22) = 39.58, *p* < .001, *ɳ*_p_^2^ = .64] as well as *SOA* [*F*(2.58, 56.75) = 89.7, *p* < .001, *ɳ*_p_^2^ = .8]. These main effects were qualified by the interaction between both factors [*F*(4.35, 95.59) = 8.11, *p* < .001, *ɳ*_p_^2^ = .27]. As can be seen from Table 1, the head-fake effect steadily increased from 2 ms at SOA 0 (*d* = 0.08) to 34 ms at SOA 300 (*d* = 1.58), and again decreased to 16 ms at SOA 800 (*d* = 0.65). Post-hoc *t*-tests were computed for the head-fake effect in dependence of SOA. Significant differences for pass and head congruent conditions as compared to incongruent conditions were found for each SOA between 100 and 800 ms (*p*s ≤ .046). No significant head-fake effect was found for SOA 0 (*p* = .695). The effect size for each SOA can be seen in Table 1. To test for significant differences of the head-fake effect between the SOAs, post-hoc paired *t*-tests for the peak (SOA 300) compared to the smallest significant head-fake effect on the left (SOA 100) and on the right (SOA 700) were conducted. The *t*-tests revealed significant differences between SOA 300 and SOA 100 [*t*(22) = 3.81, *p* = .002, *d* = 0.79], as well as for SOA 300 and SOA 700 [*t*(22) = 3.84, *p* = .002, *d* = 0.8].

**Table 1:** Mean reaction times and head-fake effect in milliseconds and effect size for each SOA.

	Pass without head fake [ms]	Pass with head fake [ms]	Head-fake effect [ms]	Effect size *d*
**SOA 0**	422	424	2.0	0.08
**SOA 100**	371	387	16.5	1.07
**SOA 200**	354	382	27.4	1.27
**SOA 300**	347	381	34.1	1.58
**SOA 400**	344	376	32.0	1.44
**SOA 500**	341	369	27.8	1.23
**SOA 600**	347	370	23.0	0.84
**SOA 700**	350	363	13.6	0.51
**SOA 800**	348	364	15.8	0.65

Interestingly, the modulation of the head-fake effect in dependence of SOA seems to be driven rather by an exponential decrease of the RTs for passes without a head fake and not by an increase of the RTs for passes with a head fake (Fig 3A). It seems like the decreasing reaction times for passes with a head fake can be described by a linear model (i.e., linear function), but the decreasing reaction times for passes without a head fake by a quadratic model (i.e., quadratic function). To further examine, which model fits the data best, curve analyses were carried out for passes with a head fake and for passes without a head fake. Since SOA 0 was different from the other SOAs (see discussion), it was excluded from these analyses. As was expected, the results for passes without a head fake did not show a significant fit of the linear model (*p* = .125, *r*^2^ = .35), but a significant fit of the quadratic model [*F*(2, 5) = 18.1, *p* = .005, *r*^2^ = .88]. For passes with a head fake, results showed a significant fit for the linear model [*F*(1, 6) = 132.96, *p* < .001, *r*^2^ = .96], and for the quadratic model [*F*(2, 5) = 65.79, *p* < .001, *r*^2^ = .96]. However, this result for the quadratic model is based on a significant result for the linear term (*p* = .019). The quadratic term of the quadratic model was not significant (*p* = .387).

### Error Rates (ER)

Mean error rates are displayed in Fig 3B. Since a Shapiro-Wilk test showed no normal distribution of the error rates (all *p*s ≤ .001), a Wilcoxon test was used to test for significant head-fakes effects for each SOA. Results showed a significant effect for SOA 200 (*z* = 3.1, *p* = .001, *r* = .65). Participants committed more errors, when a head fake was presented (2.5%) compared to passes without a head fake (0.11%). No significant head-fake effect was found for all other SOA (*p*s ≥ .059).

## Discussion

The present study investigated the optimal temporal lag between the head turn and pass initiation on the size of the head-fake effect in basketball. The aim was to provide empirical data on how to generate a large head-fake effect in the observer, in order to gain further insights into the temporal organization of deceptive actions in sports. To this end, three static images of a basketball player were subsequently presented: A basketball player in initial position (ball and head oriented towards the front), the same basketball player with the head turned to the left or right, and the same basketball player performing a pass to the left or right (with or without head fake). The SOA between the head turn and the passing action was varied. In this regard, the present study was different to previous studies, in which the head turn, and the passing action were presented (almost) simultaneously [6, 12]. As expected, a congruency effect was found, that is, faster reactions and fewer errors in pass and head congruent conditions than in incongruent conditions, signifying the well-documented head-fake effect in basketball [6]. In the reaction times, this effect was significant at all SOAs, besides SOA 0. The head-fake effect increased from SOA 0 to SOA 300 and decreased again to SOA 700. Surprisingly, this modulation of the head-fake effect seems to only be based on an overly facilitation of reactions to passes *without* a head fake and not on increased difficulties for reactions to passes with a head fake.

The results clearly show that the head turn influences reactions to a subsequent target picture. As mentioned earlier, there are different conceivable mechanisms on how the preceding head turn could affect the processing of the following passing action, namely, a shift of visual attention (i.e., cueing effect) to the side of the head turn and/or an activation of the corresponding response (i.e., priming effect). Here, we argue that the modulation of the head-fake effect seems to be induced by priming effects and possibly additional effects of attention shift. The preceding head turn activates a response corresponding to the head turn. This response activation leads to a fast response in case of a pass without a head fake. However, if the target contains a head fake, the activation of the response was incorrect and the activation of the other response must start from the beginning. Moreover, the preceding head turn could lead to an attention shift to the corresponding side. In case of a pass without a head fake, the pass direction can be processed immediately. In case of a head fake, the attention must be re-oriented to the other side before the pass direction can be processed. In this regard, Langdon and Smith [21] found priming effects as well as effects of attention shift with social cues (i.e., head and gaze cues). Based on Posner et al. [13], the authors argued that priming effects emerge earlier and lead to benefits (i.e., faster reactions in congruent conditions), whereas attention shifts need more time and cause additional costs (i.e., slower reaction in incongruent conditions) [21]. The fitting of our data to a linear and a curve model suggest that the modulation by SOA was driven by the passes without a head fake as indicated by a significant fit of the curve model. For passes with a head fake, the analysis revealed a significant fit for the linear model. Therefore, the data seem to imply that priming effects are responsible for the modulation of the effects. This view is supported by a significant head-fake effect in the error rates at SOA 200. Since this study did not contain a neutral condition, we cannot exclude an additional influence of attention shifts. However, in a preliminary study on the head-fake effect with static images, we used eye-tracking to examine possible overt visual attention shifts during the observation of head fakes. The results of 18 participants did not show any overt attention shifts, neither to the side of the head orientation nor to the pass direction. Thus, visual attention shifts might either be covert or not occur [23].

This modulation of the head-fake effect by the head turn seems to take place between SOA 200 and 600. For SOA 100, 700, and 800, we argue, that the effect is comparable to previous studies, which used single static images [6, 12]. For example, Kunde et al. [6] only used a single image of a basketball player, which either depicted a pass with or without a head fake. Based on a series of experiments, the authors assume that the head-fake effect emerges at a perceptual level. That is, responses to passes with a head fake are suggested to be slower and more error-prone than responses to passes without a head fake, as head orientation and pass direction interfere during stimulus encoding [6]. When a head-fake target appeared, participants were engaged in solving the perceptual conflict in a first step. Specifically, participants had to identify the relevant stimulus feature (i.e., pass direction) and transmit it to the stage of response selection [24]. During this process, however, no further conflict occurs, as the once identified stimulus feature (e.g., pass to the right) is always compatible with the response (e.g., right button press). Thus, for the case that head-turn and passing movement occur simultaneously (i.e., only a target image is used), the perceptual conflict covers a potential motor conflict, which occurs if the head turn precedes the passing movement, as the present results suggest. That is, the effect triggered by the head turn had not already unfold at SOA 100, but already decayed for the two longest SOA, and the effect was due to the incongruence of head and pass direction in the target [6]. In fact, the results for SOA 100, SOA 700, and SOA 800 were of similar size as those of previous studies with single static images [6, 12].

A question, which arises in this context, is, why this effect of perceptual conflict was not similarly present at SOA 0. The difference between SOA 0 and all other SOA was that no picture of the head turn alone was used at SOA 0. After the initial position of the basketball player, the target picture followed directly. Possibly, participants were not prepared to respond directly after the initial position to the target, because in 89% of the trials, the initial position was followed by the head turn. This argumentation is supported by the general reaction times, which were much larger at SOA 0 compared to all other SOAs. Therefore, participants had more time to solve the conflict, which was induced by the incongruent head orientation in the target. Moreover, a non-hypothesized result was the reduction of reaction times with increasing SOAs in general. This effect might be explained with unspecific response preparation and may be similar to the variable foreperiod effect [25, 26]. When the time interval between a warning signal and a target is uncertain (i.e., it differs from trial to trial), reaction times are shorter when the interval is long, as compared to short intervals [25].

The aim of the study was to examine at which temporal lag between the head turn and the initiation of the passing action the greatest possible head-fake effect occurs. The head-fake effect was largest for an SOA of 300 ms, However, the reaction times for passes with and without a head fake both decrease with increasing SOA (unspecific response preparation effect). Therefore, the question arises, which strategy should be recommended for sports practice. When considering the whole RT pattern, a basketball player should turn his/her head simultaneously while initiating the passing action, otherwise the full benefit of the head fake is weakened by an effect of unspecific response preparation. Simultaneous movement execution should also be much easier to implement in practice than a temporal lag of 300 ms.

This study was limited to basketball novices. However, previous studies showed that the head-fake effect can be found with novices as well as basketball experts [8], which is assumed to be based on an automatic processing of the head orientation [6]. Future studies should investigate if this result can also be replicated with basketball experts. In addition to that future studies could also focus on the deceiving athlete. Among others, interesting questions here are, if and how it is possible to train the optimal temporal organization of the head fake, and if there are any costs, which come along with performing a head fake.
